# The Goldilocks Dilemma on Balancing User Response and Reflection in mHealth Interventions: Observational Study

**DOI:** 10.2196/47632

**Published:** 2024-01-19

**Authors:** Lyndsay A Nelson, Andrew J Spieker, Lauren M LeStourgeon, Robert A Greevy Jr, Samuel Molli, McKenzie K Roddy, Lindsay S Mayberry

**Affiliations:** 1 Department of Medicine, Vanderbilt University Medical Center, Nashville, TN, United States; 2 Center for Health Behavior and Health Education, Vanderbilt University Medical Center, Nashville, TN, United States; 3 Department of Biostatistics, Vanderbilt University Medical Center, Nashville, TN, United States; 4 Department of Biomedical Informatics, Vanderbilt University Medical Center, Nashville, TN, United States

**Keywords:** engagement, mobile phone, text messaging, messaging, SMS, diabetes, diabetic, mobile health, mHealth, technology, user response, users, quality of life, engagement, mHealth management, management, socioeconomic, effectiveness, support person, support worker, support persons, text message, text messages, reflection, behavior change

## Abstract

**Background:**

Mobile health (mHealth) has the potential to radically improve health behaviors and quality of life; however, there are still key gaps in understanding how to optimize mHealth engagement. Most engagement research reports only on system use without consideration of whether the user is reflecting on the content cognitively. Although interactions with mHealth are critical, cognitive investment may also be important for meaningful behavior change. Notably, content that is designed to request too much reflection could result in users’ disengagement. Understanding how to strike the balance between response burden and reflection burden has critical implications for achieving effective engagement to impact intended outcomes.

**Objective:**

In this observational study, we sought to understand the interplay between response burden and reflection burden and how they impact mHealth engagement. Specifically, we explored how varying the response and reflection burdens of mHealth content would impact users’ text message response rates in an mHealth intervention.

**Methods:**

We recruited support persons of people with diabetes for a randomized controlled trial that evaluated an mHealth intervention for diabetes management. Support person participants assigned to the intervention (n=148) completed a survey and received text messages for 9 months. During the 2-year randomized controlled trial, we sent 4 versions of a weekly, two-way text message that varied in both reflection burden (level of cognitive reflection requested relative to that of other messages) and response burden (level of information requested for the response relative to that of other messages). We quantified engagement by using participant-level response rates. We compared the odds of responding to each text and used Poisson regression to estimate associations between participant characteristics and response rates.

**Results:**

The texts requesting the most reflection had the lowest response rates regardless of response burden (high reflection and low response burdens: median 10%, IQR 0%-40%; high reflection and high response burdens: median 23%, IQR 0%-51%). The response rate was highest for the text requesting the least reflection (low reflection and low response burdens: median 90%, IQR 61%-100%) yet still relatively high for the text requesting medium reflection (medium reflection and low response burdens: median 75%, IQR 38%-96%). Lower odds of responding were associated with higher reflection burden (*P*<.001). Younger participants and participants who had a lower socioeconomic status had lower response rates to texts with more reflection burden, relative to those of their counterparts (all *P* values were <.05).

**Conclusions:**

As reflection burden increased, engagement decreased, and we found more disparities in engagement across participants’ characteristics. Content encouraging moderate levels of reflection may be ideal for achieving both cognitive investment and system use. Our findings provide insights into mHealth design and the optimization of both engagement and effectiveness.

## Introduction

### Background

Mobile health (mHealth) is transforming health delivery as a highly convenient and effective approach for supporting individuals with chronic conditions [1-3]. Delivered via phones, tablets, and wearables, mHealth provides education, motivation, monitoring, and other forms of support to improve health behaviors. SMS text messaging is one form of mHealth that is uniquely poised to benefit everyone, including people who are older, are disadvantaged, and are from traditionally minoritized racial or ethnic backgrounds [4-6]. A critical factor influencing mHealth effectiveness is users’ engagement or interaction with the technology, which is typically measured via system use [7-9]. Across the mHealth literature, engagement tends to be highly variable [10,11], which has spurred a whole body of research that aims to understand predictors of engagement, including user characteristics and intervention features (eg, intervention duration and frequency of sending content) [10-14]. However, very little research has attended to the type of mHealth content that users are expected to engage with [15] and, more specifically, how the content may be requesting more or less cognitive reflection.

The primary goal in having users engage with mHealth content is health behavior change. With respect to mHealth interventions, there is a hyperfocus on wanting the user to interact with the technology (eg, responding to a text message), with less consideration of whether the user is reflecting on the content cognitively (eg, reflecting on past behavior and planning future behavior) [8]. Although interaction with the technology is a critical measure, there is a growing consensus that cognitive investment is also important for meaningful behavior change in many types of mHealth interventions [16-18]. Notably, content may be designed in a way that represents a low response burden, thereby easily eliciting a response (ie, producing high engagement), but such content may not evoke the necessary cognitive reflection required to change behavior [18]. Alternatively, content that is designed to encourage deeper reflection may overwhelm users, which risks them disengaging completely. Understanding how to strike the balance between response and reflection has critical implications for effective engagement (ie, engagement needed to impact outcomes) [19]. To our knowledge, no studies have explored the association between reflection demands and the degree of interaction with an mHealth tool. Understanding the interplay between reflection burden and response burden will help guide the design of interventions seeking to strike this balance.

### Objective

Our team previously developed an mHealth intervention (delivered via text messages and phone calls) called *Family/Friend Activation to Motivate Self-care* (FAMS) [20,21]. FAMS is a diabetes self-management intervention that targets persons with type 2 diabetes and provides the option for persons with diabetes to invite a support person to also receive text messages. We recently evaluated FAMS in a randomized controlled trial (RCT) [22], and during routine monitoring in the first few weeks of the trial, we observed a low response rate to text messages among support persons. Because support persons’ engagement with the text messages was an optional component of the intervention, we determined that this was an opportunity to explore how changing the content of these texts might impact response rates without compromising our ability to evaluate FAMS’ effects. Over the course of the RCT [22], we used a pragmatic approach to vary both the reflection burden and the response burden of the two-way text messages sent to support persons assigned to the intervention. In this observational study, our primary goal was to explore how these variations would impact users’ engagement with text messages, as measured via response rates. We also explored support persons’ characteristics that were associated with response rates for each type of text and described the different responses to each text.

## Methods

### Study Design and Eligibility

This study was conducted as part of the FAMS 2.0 RCT. The trial design, intervention details, and outcomes for persons with diabetes and support persons were published [22-24]. For the trial, dyads comprising a person with diabetes and their support person were randomized to FAMS or a control condition. We recruited persons with diabetes who were receiving care for type 2 diabetes at Vanderbilt University Medical Center primary care clinics. Enrolling persons with diabetes were asked to invite a support person to participate with them and receive text messages; however, support person invitation and enrollment were not required. We defined a *support person* as any family member or friend with whom the person with diabetes would feel comfortable talking about diabetes management and health goals. Eligible support persons were aged ≥18 years, could speak and read English, and had a mobile phone separate from that of the person with diabetes. The only exclusion criterion was the inability to receive and respond to a text after training. For this study, we analyzed data from support persons in dyads that were randomly assigned to the intervention group (FAMS).

### Ethical Considerations

The Vanderbilt University Institutional Review Board approved all study procedures (institutional review board number: 200398; approved April 8, 2020), and the trial was registered on ClinicalTrials.gov (trial number: NCT04347291).

#### Procedure

While enrolling persons with diabetes into the trial, research assistants collected contact information for a potential support person. A research assistant then contacted potential support persons to verify interest and eligibility, obtain verbal informed consent, and ask support persons to complete a baseline survey. Surveys were completed by phone with a research assistant, on the web via an emailed link, or via a mailed paper copy, per participants’ preferences. All survey data were stored in REDCap (Research Electronic Data Capture; Vanderbilt University), a secure web-based platform that supports data capture for research studies [25,26]. In addition to collecting data on sociodemographic characteristics, surveys asked support persons to choose the time of day they wanted to receive text messages. Relevant survey responses were transferred from REDCap to our technology partner, PerfectServe, using an automated application programming interface. PerfectServe used participant information to tailor, schedule, and send text messages to support persons for 9 months. Support persons could earn a total of US $120 for completing all study surveys (through 15 mo for the larger RCT). There was no compensation for receiving or responding to text messages.

### The Intervention

Persons with diabetes received daily text message support and monthly coaching sessions, during which they set behavioral diabetes self-management goals (as detailed by Mayberry et al [22]). Support persons received text messages that were designed to increase dialogue about and facilitate their involvement in the diabetes self-management of the persons with diabetes; a one-way message was sent 3 to 4 times per week, and a two-way message (also known as an *interactive text message*) was sent once per week. One-way messages were either a general text message about providing diabetes self-management support or a text message tailored to the identified diabetes goals of the persons with diabetes. Two-way messages asked support persons about how they supported the health of the persons with diabetes. Support persons who replied to the two-way text received an automated response, thanking them for their answer.

**Figure 1.**
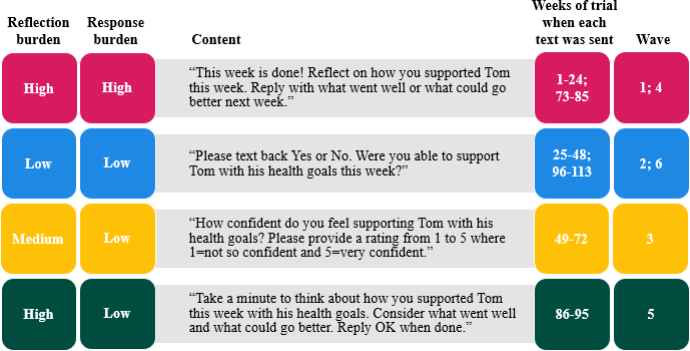
The four versions of the two-way text message. The content references an example person with diabetes named Tom.

Although an individual support person’s intervention experience lasted 9 months (36 wk), intervention delivery for the trial lasted 2 years. Over the course of those 2 years, we varied both the response burden (the level of information requested for the response relative to that of other messages) and the reflection burden (the level of cognitive reflection requested relative to that of other messages) of the weekly two-way text messages, which were sent to support persons in 6 fixed periods (ie, waves). The waves coincided with the weeks of the trial; they did not coincide with the weeks of each individual support person’s intervention experience. Figure 1 includes the content for each version of the text message, the weeks of the trial when each text was sent (ie, calendar time), and the respective waves. We started the trial (wave 1) by sending a text message that was high in both reflection burden and response burden (*high/high*). In wave 2, we tested a text that was low in both reflection burden and response burden (*low/low*), and then in wave 3, we tested a text that involved medium reflection burden and low response burden (*medium/low*). In wave 4, we retested the *high/high* message to help determine if the point at which the text was sent during the trial impacted engagement. In wave 5, we sought to delineate the relative impacts of reflection burden and response burden; therefore, we tested a text message that was high in reflection burden and low in response burden (*high/low*). Finally, we closed out the trial by retesting the *low/low* text message (wave 6). The decisions about what messages to test were made iteratively based on response rates to the prior message, with the goal of learning how much reflection we could request while still achieving a relatively high response rate.

Of note, each support person only received the versions of the two-way text message that were sent during their 36-week trial participation, with most (120/148, 81.1%) receiving 2 or 3 different versions and no participants receiving the same message in 2 separate waves. Because this analysis used data from support persons only, we refer to them as *participants* henceforth.

### Measures

#### Sociodemographic and Relationship Characteristics

We collected self-reported data on age, gender, race, ethnicity, socioeconomic status (measured based on education [ie, years in school] and annual household income), and health literacy (assessed via the Brief Health Literacy Screen [27]). In addition, we asked whether participants were cohabitating with the persons with diabetes and the frequency with which they provided diabetes-specific helpful involvement to the persons with diabetes at baseline, as assessed via the Family and Friend Involvement in Adults’ Diabetes (FIAD) helpful subscale, support person version [28].

#### Engagement

We operationalized engagement by using response rate (ie, two-way messages responded to divided by the two-way messages sent, for each participant).

### Analyses

#### Statistical Analysis Overview

All statistical analyses were performed by using R version 4.2.1 (R Foundation for Statistical Computing). We described participant characteristics via means and SDs or via frequencies and percentages, as appropriate. Except for when examining temporality, message waves that included the same version of the two-way text message were grouped together. Because this study was exploratory, we did not perform sample size calculations.

#### Overall Engagement by Text Message Version

For each version of the text message, we determined the proportion of two-way text messages sent to support persons that received a response by study week (ie, calendar time). We also generated summary statistics (means, medians, and first and third quartiles) for response rates at the participant level; reporting both mean and median provides more detailed information on the distribution of data. If participants withdrew during their intervention experience, we calculated their response rates based on the data available prior to their withdrawal. To account for repeated measures within participants, we used generalized estimating equations with a working independence correlation structure and a logistic link function to compare the odds of responding to two-way text messages across the four versions.

#### Participant Characteristics and Engagement

We used Poisson regression to estimate associations (as incidence rate ratios) between participant characteristics and text message response rates for each version of the text message. We included the number of two-way messages sent to a participant as an offset term in order to account for variation in the number of messages sent to each participant in a given wave; therefore, the exponentiated coefficients from the Poisson regression model compared response rates on a per-message basis. Participant characteristics included age, race and ethnicity (non-Hispanic White vs minoritized race or ethnicity), gender, education (years), annual household income (≥US $50,000 per year), health literacy (Brief Health Literacy Screen), whether the persons with diabetes and support persons were cohabitating, and self-reported baseline helpful involvement (FIAD). Further, we multiplied participants’ age by 10 to allow for easier interpretation of the results. Especially in regression models, it can be difficult to interpret the association between age and an outcome when the change in the outcome is based on a single-year change in age (ie, the coefficients end up being too small). Scaling the age variable in this way allowed us to interpret the findings in a more meaningful way, that is, we compared groups that differed in age by 1 decade rather than 1 year.

For this analysis, we excluded 5 participants who were missing all baseline data. However, missing covariate values were otherwise addressed via multiple imputation by chained equations (*M*=500 iterations).

#### Types of Responses

We characterized the responses to each version of the two-way text message. For the *low/low*, *medium/low*, and *high/low* texts, we reported the frequency of responses based on what the respective text requested (eg, “Yes,” “No,” “1,” “2,” “3,” “4,” or “ 5”). For the *high/high* text messages, 2 team members reviewed responses and categorized each as being either high effort or low effort. High-effort responses included comments on what went well that week, comments on what could go better next week, or both, and they referred to a diabetes self-management behavior such as diet, exercise, stress management, or communication (eg, “[He] and I got out several times this week walking after work. Our biggest problem is watching portion size when we are eating. Always continue to work on that.”). A low-effort response consisted of only a brief phrase that did not reference a diabetes self-management behavior (eg, “Things went well” and “We were on vacation this week”) or did mention a behavior but was unclear as to what went well or what could go better next week (eg, “Walking”).

## Results

### Participant Characteristics

In the trial, of the 150 support person participants who were enrolled and randomized to receive the FAMS intervention, 2 withdrew before the intervention started. The remaining 148 were included in the analyses (Table 1). The mean age was 50.3 (SD 14.7) years; 28.4% (42/148) of participants were men, and 33.1% (49/148) reported a minoritized racial or ethnic background. The mean length of education was 14.9 (SD 2.5) years, and 31.1% (46/148) of participants had annual household incomes of <US $50,000. Over half (84/148, 56.8%) were spouses or partners of the persons with diabetes, and 70.3% (104/148) were cohabitating with the persons with diabetes. Further, 9 participants withdrew at some point during the intervention; the analyses below reflect their engagement during the time they participated.

**Table 1.** Participant characteristics (N=148).

Characteristic			Value
Age^a^ (y), mean (SD)			50.3 (14.7)
**Gender** ^**b**^ **, n (%)**			
	Men		42 (28.4)
	Women		101 (68.2)
**Race and ethnicity, n (%)**			
	Non-Hispanic White		92 (62.2)
	Non-Hispanic Black		29 (19.6)
	Other non-Hispanic races		12 (8.1)
	Hispanic		8 (5.4)
	Missing		7 (4.7)
**Socioeconomic status**			
	Education^c^ (y), mean (SD)		14.9 (2.5)
	**Annual household income (US $), n (%)**		
		<35,000	25 (16.9)
		35,000-49,999	21 (14.2)
		50,000-74,999	22 (14.9)
		75,000-99,999	24 (16.2)
		≥100,000	40 (27)
		Missing or unknown	16 (10.8)
	Health literacy (BHLS^d^^,^^e^), mean (SD)		13.7 (1.5)
**Relationship variables**			
	**Relationship type** **, n (%)**		
		Spouse or partner	84 (56.8)
		Parent	15 (10.1)
		Son or daughter	22 (14.9)
		Grandchild	7 (4.7)
		Friend	11 (7.4)
		Other	4 (2.7)
		Missing	5 (3.3)
	Helpful involvement (FIAD^f^^,^^g^), mean (SD)		2.7 (0.9)
	Cohabitating with person with diabetes^h^, n (%)		104 (70.3)

a 10 participants did not report their date of birth (ie, age).

b 5 participants did not provide data on gender.

c 8 participants did not report years of education.

d BHLS: Brief Health Literacy Screen.

e 5 participants did not have data for the BHLS measure.

f FIAD: Family and Friend Involvement in Adults’ Diabetes.

g 5 participants did not have data for the FIAD helpful subscale.

h 8 participants did not have data about cohabitating with the persons with diabetes.

### Overall Engagement by Text Message Type

**Figure 2.**
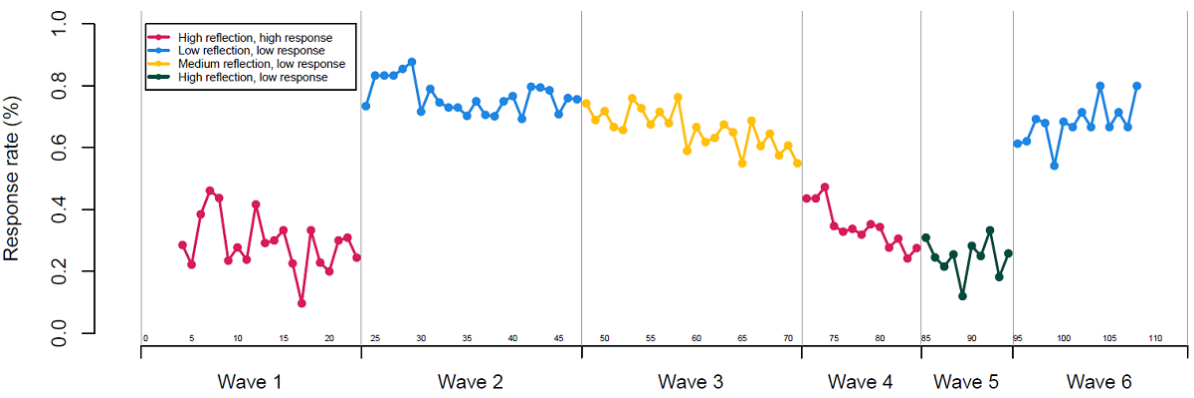
Text message response rates by week across each wave. Response data were excluded for the first 4 weeks and the last 5 weeks of the trial when <5 individuals were receiving the intervention.

Figure 2 presents the proportion of two-way text messages that received a response within each week of the trial (ie, by calendar time). Notably, text message response rates for waves 1 and 4 (both were *high/high* message waves) were comparable, as were those for waves 2 and 6 (both were *low/low* message waves). Table 2 includes descriptive statistics for the overall and participant-level response rates for each version of the two-way text. The median response rates for the *high/high*, *medium/low*, and *low/low* messages were 23% (IQR 0%-51%), 75% (IQR 38%-96%), and 90% (IQR 61%-100%), respectively. When we kept reflection burden high but lowered response burden in the *high/low* message, the median response rate (10%, IQR 0%-40%) was closest to that for the *high/high* message, suggesting that reflection burden was responsible for the lower response rates seen with the *high/high* message.

**Table 2.** Response rates for each version of the two-way text message.

Reflection burden and response burden	Participants included in analysis, n	Participant-specific response rate	
		%, mean (SD)	%, median (IQR)
High and high	127	33 (33)	23 (0-51)
Low and low	123	74 (32)	90 (61-100)
Medium and low	125	65 (35)	75 (38-96)
High and low	55	26 (32)	10 (0-40)

We also compared the odds of responding to the four versions of the text message (Table 3). When compared to the *high/high* message, the odds of responding to the *low/low* message was 53% (95% CI 42%-65%) higher, the odds of responding to the *medium/low* message was 40% (95% CI 31%-49%) higher, and the odds of responding to the *high/low* message was 7.5% (95% CI 0.7%-14%) lower. All other pairwise comparisons (Table 3) indicated decreasing odds of responding at increasing levels of reflection burden.

**Table 3.** Comparison of text message response rates by text message version. Included are odds ratios (ORs) and 95% CIs, along with *P* values.

Comparison		OR (95% CI)	*P* value
**Relative to high reflection burden and high response burden**			
	Low reflection burden and low response burden	1.53 (1.42-1.65)	<.001
	Medium reflection burden and low response burden	1.40 (1.31-1.49)	<.001
	High reflection burden and low response burden	0.93 (0.86-0.99)	.03
**Relative to low reflection burden and low response burden**			
	Medium reflection burden and low response burden	0.92 (0.86-0.98)	.01
	High reflection burden and low response burden	0.60 (0.55-0.66)	<.001
**Relative to medium reflection burden and low response burden**			
	High reflection burden and low response burden	0.66 (0.61-0.72)	<.001

### Participant Characteristics Associated With Odds of Responding

Table 4 presents estimated incident rate ratios, along with 95% CIs and *P* values, from multivariate Poisson regression models that were used to identify participant characteristics predictive of response rate. Younger participants, participants who were not cohabitating with the persons with diabetes, and participants who had a lower socioeconomic status had lower response rates to both the *high/high* message and the *medium/low* message compared to those of older participants, participants who were cohabitating, or participants who had a higher socioeconomic status, respectively. The only characteristic associated with response rates for the *low/low* message was gender, such that men had lower response rates. Further, the only characteristic associated with response rates for the *high/low* message was age, such that younger age was associated with lower response rates. Across message versions, younger participants had lower response rates to any message with more burden than the *low/low* message, and participants who were not cohabitating with the persons with diabetes had lower response rates to the higher-burden messages than those of participants who were cohabitating. Race, ethnicity, health literacy, and baseline helpful involvement provided to the person with diabetes did not show patterns indicating the prediction of response rates to any message version.

**Table 4.** Participant characteristics predicting text message response rates for each version of the text. Presented are estimated incident rate ratios (IRRs) and 95% CIs, along with *P* values.^a^

Predictor	High reflection/high response		Low reflection/low response		Medium reflection/low response		High reflection/low response	
	IRR (95% CI)	*P* value	IRR (95% CI)	*P* value	IRR (95% CI)	*P* value	IRR (95% CI)	*P* value
Age (y × 10)	1.14 (1.06-1.23)	.004^b^	0.99 (0.95-1.03)	.67	1.08 (1.04-1.14)	.001^b^	1.28 (1.09-1.51)	.002^b^
Race and ethnicity	1.07 (0.85-1.35)	.57	0.96 (0.84-1.09)	.51	0.94 (0.81-1.09)	.40	0.60 (0.36-1.01)	.052
Gender (men)	0.62 (0.49-0.78)	<.001^b^	0.78 (0.68-0.89)	<.001^b^	0.89 (0.78-1.02)	.10	1.28 (0.83-1.98)	.26
Education	0.98 (0.94-1.02)	.34	1.00 (0.98-1.03)	.77	0.96 (0.94-0.99)	.007^b^	0.99 (0.90-1.09)	.87
Income	0.71 (0.57-0.89)	.003^b^	1.05 (0.91-1.21)	.51	1.14 (0.98-1.33)	.10	0.77 (0.46-1.27)	.30
BHLS^c^	1.04 (0.97-1.12)	.30	1.04 (1.00-1.09)	.06	1.02 (0.98-1.06)	.24	1.02 (0.86-1.21)	.80
Cohabitating	1.37 (1.09-1.73)	.007^b^	1.03 (0.90-1.19)	.64	1.21 (1.05-1.40)	.008^b^	1.63 (0.93-2.85)	.09
FIAD^d^	1.05 (0.94-1.17)	.41	1.00 (0.93-1.07)	.99	0.99 (0.93-1.06)	.79	0.90 (0.71-1.14)	.37

a A total of 5 support persons without baseline characteristics were excluded from this analysis: 2 were excluded from the models for the *high/high*, *low/low*, and *medium/low* messages; 1 was excluded from the models for the *high/high* and *medium/low* messages; 1 was excluded from the models for the *high/high*, *low/low*, and *high/low* messages; and 1 was excluded from the models for the *low/low* and *medium/low* messages.

b *P*<.05.

c BHLS: Brief Health Literacy Screen.

d FIAD: Family and Friend Involvement in Adults’ Diabetes.

### Types of Responses

In this section, we report on engagement at the text message level (vs the participant level). For the *high/high* messages, 1429 texts were sent, and 445 responses were received. Further, 13 responses were excluded from the analysis because the content was uninterpretable or was not relevant to the two-way text prompt. The reviewers categorized each response into the high- or low-effort response category, with 98.6% (responses: 426/432) agreement; of the 426 texts agreed upon, 350 (82.2%) were categorized as high-effort responses, and 76 (17.8%) were categorized as low-effort responses. For the *low/low* texts, 1791 texts were sent, and 1341 responses were received; almost all of the responses were “yes” responses (n=1239, 92.4%), while only 97 (7.2%) were “no” responses, and 5 (0.4%) were considered “other” responses. For the *medium/low* texts, 1847 texts were sent, and 1218 responses were received; the most common response was a “5” response (n=681, 55.9%), followed by a “4” response (n=338, 27.8%) and then a “3” response (n=144, 11.8%). Lastly, for the *high/low* texts, 446 texts were sent, and 109 responses were received; most of the responses received were “OK” responses, as requested in the message (n=86, 78.9%).

## Discussion

### Principal Results

Despite the potential of mHealth to enhance self-management support and quality of life, there are still key gaps in understanding how to optimize mHealth engagement [16,17,19]. Most engagement research reports only on system use without consideration of the cognitive reflection done in the process of engaging with the content [16,29]. Ideally, we want to encourage reflection that results in meaningful behavior change, but it is unclear how much we can request, with respect to reflection, before users disengage. We varied the reflection and response burdens of two-way text messages to examine how these variations impacted users’ engagement, as assessed via response rates. We found, generally, that as the reflection burden of the message increased, participants’ engagement decreased. Importantly, when the same version of the text was sent at different points in the trial, participants’ engagement was consistent, suggesting that the message itself was key for response rates. The response rates for the *high/low* message were similar to those for the *high/high* message, and this supports reflection burden (vs response burden) being the primary driver of lower engagement. We also found evidence that as the reflection burden of the message increased, there were more disparities in engagement across participant characteristics. This finding helps inform who we may lose with content requiring more from users and thus how to ensure mHealth interventions do not widen existing health disparities.

Research focused on promoting mHealth engagement has proliferated in recent years, with the primary goal of increasing system use [30]. Across such studies, we see evidence that content that is more personalized; uses simpler, nontechnical language; and is empowering results in higher engagement [10,19,31-35]. Less research has compared specific types of content and has rarely tested different types within the same study. An exception is a recent study by Klimis et al [15], wherein they used machine learning to demonstrate that text messages with informative (providing health facts or education) and instructional (providing tips or recommendations) message intents were associated with increased engagement, while notification messages that addressed noneducational matters (eg, welcome and exit messages) were associated with reduced engagement [15]. Our study targeted a two-way message and varied the levels of reflection and response burdens in that message. By adjusting the reflection level specifically, we gained unique insight into how engagement with each message variation may ultimately influence behavior change [17,18]. The main way in which our study differs from others in this area of research is that we looked beyond system use as the sole dimension of mHealth engagement. Our goal was not necessarily to see which message resulted in the highest response rate but rather to determine how much reflection we could request from users and achieve a level of interaction that suggested that they were still invested in the content.

Although our results show generally that engagement decreases with more reflection, the nuances in our findings allow us to provide unique recommendations around mHealth design. For instance, it may be best to alternate through content with different levels of reflection burden. Although users were more likely to respond to content that was lower in reflection burden, nearly all (350/426, 82.2%) of the responses that we received to the high-reflection messages included a high-effort level of reflection. The act of asking people to reflect stimulates internal thoughts that are difficult to measure without a response [36] but may still occur among some persons who do not respond. Alternating content may help promote periodic responding and reflecting throughout an intervention experience. Another option involves using an adaptive intervention to tailor the content based on each person’s responsiveness. That is, everyone could start receiving content with a high reflection burden, but if a person’s response rate starts to drop, they could then switch to content with a moderate reflection burden. Finally, especially in situations where there is limited flexibility with the mHealth functionality, researchers may consider sending the *medium/low* message to all participants, given that the content encouraged a moderate level of reflection (more than the *low/low* message) yet still yielded a high response rate.

### Limitations

Our study has several limitations to acknowledge. For instance, our results are based on an SMS text messaging intervention, which is a specific form of mHealth. It is possible that users would have responded differently if the content was delivered via an app or wearable technology. Importantly, compared to apps and other internet-dependent technologies, SMS text messaging is both lower in cost and more easily accessed, and it tends to have higher rates of engagement [4,37]. In addition, this study recruited persons with diabetes and their support persons from a specific region in Middle Tennessee. We acknowledge that the findings may not be generalizable to other types of individuals who are living in other locations. Relatedly, the content asked about how the support persons supported the health of the persons with diabetes, and engagement may differ when asking about a user’s own health; however, the marked differences in engagement across message types support broader applications. Another limitation of our work is that we restricted our assessment of engagement to a behavioral measure (ie, responding to the text) and did not have a way to assess participants’ cognitive investment or experience with each version of the text. Based on our analysis of participants’ responses to the *high/high* message, it appeared that responders were cognitively engaged, but we were not able to compare cognitive engagement across the other messages. In addition, the sample size for the *high/low* message analysis (n=55) was considerably smaller compared to those for the other message analyses, which was due to testing the *high/low* message during only 1 wave near the end of the trial when fewer participants were enrolled. Relatedly, we did not have the time or a sufficient number of participants toward the end of the trial to test the *medium/low* and *high/low* text messages in a second wave, and we do not know for certain whether engagement with these texts could be impacted by temporality; however, as engagement with the *high/high* and *low/low* texts remained similar across multiple waves, it is unlikely. The ordering of the text messages was variable across participants, and due to the observational nature of this study, we cannot determine the extent that ordering may have impacted results; however, the average response rates and trends across message versions and waves provide general insights on how these variations may impact engagement. Finally, we did not assess the impact of engagement on outcomes, as this fell outside the scope of our study; however, other studies in digital health have examined this association [38,39].

### Conclusions

In order for individuals to benefit from mHealth and achieve desired effects on outcomes, engagement with the mHealth tool is needed. Our results help elucidate how truly complex the nature of engagement is. Although our past work and that of others have demonstrated the importance of behaviorally interacting with mHealth interventions [9,31,40,41], this measure represents one piece of a larger puzzle. Engagement may be best conceptualized as including both a behavioral dimension and a cognitive dimension. Balancing these dimensions may be what is ultimately needed to achieve effective engagement for impacting intended outcomes. Our study contributes to a growing body of research that encourages a more nuanced approach to studying engagement that goes beyond measuring system use. We hope that our findings help advance the field of mHealth and inform intervention design, with the goal of optimizing both engagement and effectiveness.

## Abbreviations

FAMS: Family/Friend Activation to Motivate Self-care
FIAD: Family and Friend Involvement in Adults' Diabetes
mHealth: mobile health
RCT: randomized controlled trial
REDCap: Research Electronic Data Capture
